# Impact of Fast Sodium Channel Inactivation on Spike Threshold Dynamics and Synaptic Integration

**DOI:** 10.1371/journal.pcbi.1001129

**Published:** 2011-05-05

**Authors:** Jonathan Platkiewicz, Romain Brette

**Affiliations:** 1 Laboratoire Psychologie de la Perception, CNRS and Université Paris Descartes, Paris, France; 2 Département d'Etudes Cognitives, Ecole Normale Supérieure, Paris, France; Université Paris Descartes, Centre National de la Recherche Scientifique, France

## Abstract

Neurons spike when their membrane potential exceeds a threshold value. In central neurons, the spike threshold is not constant but depends on the stimulation. Thus, input-output properties of neurons depend both on the effect of presynaptic spikes on the membrane potential and on the dynamics of the spike threshold. Among the possible mechanisms that may modulate the threshold, one strong candidate is Na channel inactivation, because it specifically impacts spike initiation without affecting the membrane potential. We collected voltage-clamp data from the literature and we found, based on a theoretical criterion, that the properties of Na inactivation could indeed cause substantial threshold variability by itself. By analyzing simple neuron models with fast Na inactivation (one channel subtype), we found that the spike threshold is correlated with the mean membrane potential and negatively correlated with the preceding depolarization slope, consistent with experiments. We then analyzed the impact of threshold dynamics on synaptic integration. The difference between the postsynaptic potential (PSP) and the dynamic threshold in response to a presynaptic spike defines an effective PSP. When the neuron is sufficiently depolarized, this effective PSP is briefer than the PSP. This mechanism regulates the temporal window of synaptic integration in an adaptive way. Finally, we discuss the role of other potential mechanisms. Distal spike initiation, channel noise and Na activation dynamics cannot account for the observed negative slope-threshold relationship, while adaptive conductances (e.g. K+) and Na inactivation can. We conclude that Na inactivation is a metabolically efficient mechanism to control the temporal resolution of synaptic integration.

## Author Summary

Neurons spike when their combined inputs exceed a threshold value, but recent experimental findings have shown that this value also depends on the inputs. Thus, to understand how neurons respond to input spikes, it is important to know how inputs modify the spike threshold. Spikes are generated by sodium channels, which inactivate when the neuron is depolarized, raising the threshold for spike initiation. We found that inactivation properties of sodium channels could indeed cause substantial threshold variability in central neurons. We then analyzed in models the implications of this form of threshold modulation on neuronal function. We found that this mechanism makes neurons more sensitive to coincident spikes and provides them with an energetically efficient form of gain control.

## Introduction

Action potentials are initiated when the membrane potential exceeds a threshold value, but this value depends on the stimulation and can be very variable *in vivo* [1]–[4], which has triggered a recent controversy about the origin of this variability [5]–[7]. This phenomenon has been observed in many areas of the nervous system: visual cortex [1]–[3], somatosensory cortex [4]; prefrontal cortex [8]; neostriatum [9], neocortex [10], [11], hippocampus [12], [13], and auditory brainstem [14]–[17]. Experimental studies have shown that the spike threshold is correlated with the average membrane potential [2], [8], inversely correlated with the preceding rate of depolarization [1]–[4], [9], [12], [14] and inversely correlated with the preceding interspike interval [13], [18]. Thus, threshold dynamics participate in the input-output properties of neurons: it enhances coincidence detection and gain modulation properties [1], [2], it contributes to feature selectivity in sensory processing [2], [4], [19], contrast invariance [2], [20] and temporal coding [17], [21], [22].

Among the mechanisms that can modulate the spike threshold [23], two are thought to be especially relevant: inactivation of sodium channels [1], [2], [4], [8], [12], [17] and activation of potassium channels [2], [10]–[12], [14]–[16]. In this study, we chose to focus on the role of sodium channel inactivation because it specifically impacts spike initiation without changing the membrane potential, and because of the extensive voltage-clamp data available for Na channels. Our first goal was to check whether Na channel inactivation, given their measured properties, can account for significant threshold variability and for the qualitative properties of the spike threshold dynamics, as listed above. Our second goal was to evaluate the consequences of threshold dynamics on the integration of postsynaptic potentials (PSPs).

We analyzed the influence of Na inactivation on spike threshold in a model, in which we were able to express the spike threshold as a function of Na channel properties and variables [23]. We collected previously published voltage clamp measurements of Na channel properties and found that Na inactivation by itself can account for substantial threshold variability, with the same qualitative properties as experimentally observed. To investigate the implications for synaptic integration, we derived a dynamical equation for the spike threshold and defined effective PSPs as the difference between the PSP and the threshold. We found that, with threshold adaptation as implied by Na inactivation, effective PSPs are briefer than PSPs and that their shape depends on membrane depolarization. Finally, we discuss the potential contribution of other mechanisms of threshold modulation.

## Results

### The threshold equation

We previously derived a formula, the threshold equation, which relates the instantaneous value of the spike threshold to ionic channels properties [23]:
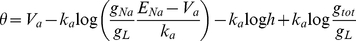
where V_a_ is the half-activation voltage of Na channels, k_a_ is the activation slope factor, g_Na_ is the total Na conductance, g_L_ is the leak conductance, E_Na_ is the Na reversal potential, h is the inactivation variable (1-h is the fraction of inactivated Na channels). Here the spike threshold is defined as the voltage value at the minimum of the current-voltage function in the membrane equation (we compared various threshold definitions in [23]). This formula is derived from the assumption that the Na activation curve is well described by a Boltzmann function, which implies that the Na current below spike initiation is close to an exponential function of voltage (see Text S1 for the derivation). This approximation of the Na current is the basis of the exponential integrate-and-fire model (EIF) [24]. In this paper, we focus on the impact of Na inactivation and therefore we ignore the last term of the threshold equation, which simplifies to:
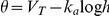
where V_T_ is a constant term, corresponding to the minimum spike threshold (when Na channels are not inactivated). We call the EIF model with Na inactivation the inactivating exponential integrate-and-fire model (iEIF; see Methods). After a spike, the voltage is reset to the resting potential E_L_, and *h* is unchanged. Thus, when the neuron is depolarized, Na channels inactivate (h decreases) and the threshold increases: the threshold adapts to the membrane potential.

### Steady-state threshold and threshold variability

**Figure 1:**
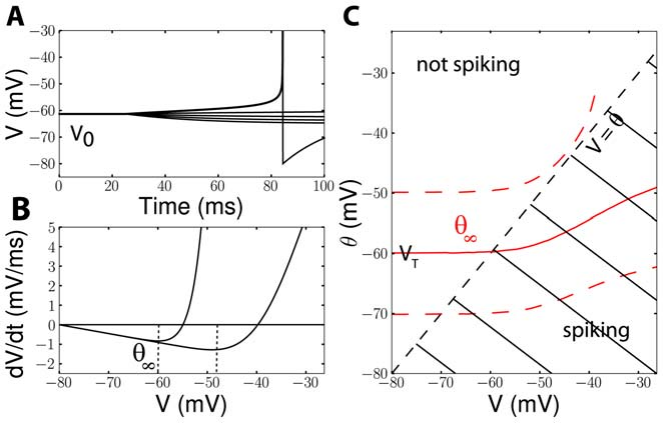
Steady-state threshold. A, The membrane potential is clamped at a given voltage 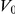, then a constant current I is injected (iEIF model). The steady-state threshold 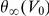 is defined as the maximum voltage that can be reached without triggering an action potential. B, Two excitability curves dV/dt = F(V,V_0_)/C are shown in the phase plane 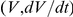, for two different initial clamp values V_0_ (solid lines; V_0_ = −80 mV and −26 mV). The steady-state threshold 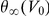 is the voltage at the minimum of the excitability curve for the initial voltage V_0_. C, Steady-state threshold (red lines) of a cortical neuron model [63] for the original maximal Na conductance (solid line) and for a higher and lower Na conductance (resp. bottom and top dashed line). When the cell is slowly depolarized, it spikes when 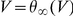, i.e., the spike threshold is the intersection of the red and black dashed curves. If there is no intersection, the neuron cannot spike with slow depolarization. The top dashed line (low Na conductance) is interrupted because the threshold is infinite at high voltages (i.e., the cell is no longer excitable).

We start by studying the steady-state threshold, which is the value 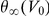 of the spike threshold for a fixed voltage V_0_. It corresponds to the threshold measured with the following experiment. The cell is clamped at a voltage V_0_ (Figure 1A), and a fraction of Na channels inactivates. In the Hodgkin-Huxley formalism, this fraction is 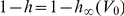, where 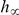 is the steady-state inactivation function (h is the fraction of non-inactivated channels). If the clamp is relaxed and a current is injected, the neuron may produce a spike if the current is large enough (Figure 1A). The steady-state threshold 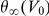 corresponds to the maximum voltage that can be reached without triggering an action potential, and it depends on the fraction (1-h) of inactivated Na channels: when the membrane is depolarized, Na channels inactivate, which raises the spike threshold.

One way to understand threshold adaptation is to look at how the excitability curve changes with h (and therefore with depolarization). The excitability curve (Figure 1B) shows the value of dV/dt vs. V for a fixed value of h, as given by the membrane equation (which is equivalent to the I-V curve, if the current is scaled by the membrane capacitance). When h decreases (Na channels inactivate), the entire excitability curve shifts towards higher voltages and the threshold shifts accordingly. As in [23], we define the threshold as the voltage at the minimum of the excitability curve, but since the entire curve is shifted by Na inactivation, other definitions would produce similar results.

The membrane potential V is always below threshold, unless the cell spikes. Therefore the observable threshold values cannot be larger than the intersection between the threshold curve and the diagonal line 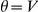, if these two curves intersect (Figure 1C). Thus, the spike threshold may vary between the minimum steady-state threshold V_T_ and the solution of 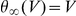. When there is no such solution, the threshold can be arbitrarily large, meaning that a very slow depolarization would not elicit a spike (Figure 1C, top dashed curve). Thus, the range of threshold variability can be derived from the steady-state threshold curve.

**Figure 2:**
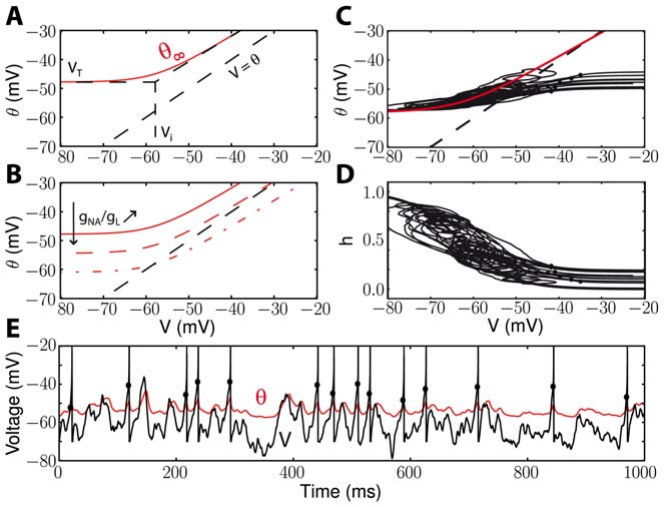
Role of Na channel properties in threshold variability in the iEIF model. A, The steady-state threshold curve (red curve) is well approximated by a piecewise linear curve determined by Na channel properties (top dashed black curve), where V_i_ is the half-inactivation voltage and V_T_ is the non-inactivated threshold. The slope of the linear asymptote is k_a_/k_i_ (resp. activation and inactivation slope parameters). Na channel properties in this figure were taken from Kuba et al. (2009). The spike threshold is variable only when 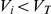, and very variable when (additionally) 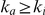. B, The non-inactivated threshold V_T_ is determined by the maximum Na conductance g_Na_, relative to the leak conductance g_L_. As the ratio 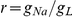 increases, the steady-state threshold curve 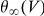 shifts downward (red curves; r = 0.4; 2; 10) and threshold variability is reduced. C, Trajectory of the model in the 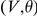 phase plane (blue), superimposed on the steady-state threshold curve (red). Spikes are initiated when 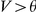 (dashed line: 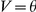), but the empirical measurement overestimates the threshold. The spike threshold is highly variable in this example (−50 to −10 mV). D, Trajectory of the model in the 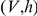 phase plane (blue), superimposed on the Na inactivation curve (black). The threshold is very variable when most Na channels are inactivated. E, Voltage trace (black curve) and spike threshold 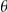 (red curve; 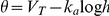) in the inactivating exponential model driven by a fluctuating input (see Methods), where black dots represent empirical measurement of spike onsets (first derivative method, k_th_ = 5 mV/ms). Note that the membrane potential can exceed threshold without triggering a spike because the threshold is soft (unlike in integrate-and-fire models).

Using the threshold equation, we can calculate the steady-state threshold as a function of V: 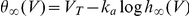, where 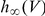 is the Na inactivation curve, which is generally well fitted by a Boltzmann function [25]:
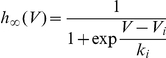
where 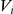 is the half-inactivation voltage, and 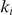 is the inactivation slope factor. When we substitute this function in the threshold equation, we find that the steady-state threshold has a horizontal asymptote (V_T_) for large negative potentials and a linear asymptote for large positive potentials, because the inactivation function is close to exponential (Figure 2A). Thus, the steady-state threshold can be approximated by a piecewise linear function (see Text S1):
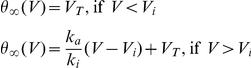



In other words, the minimum threshold is V_T_, which is determined by the maximum Na conductance (Figure 2B), the threshold increases above the half-inactivation voltage V_i_, and the slope is the ratio of activation and inactivation slope factors. Regarding threshold variability, we can distinguish three cases, depending on Na channel properties:

1. if 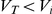 then the spike threshold is constant 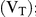

2. if 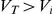 and 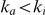, then the threshold varies between 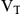 and 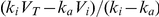;
3. if 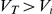 and 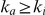, then the threshold can be arbitrarily large (that is, the neuron can be continuously depolarized without triggering spikes, as observed in some preparations [26]).

**Figure 3:**
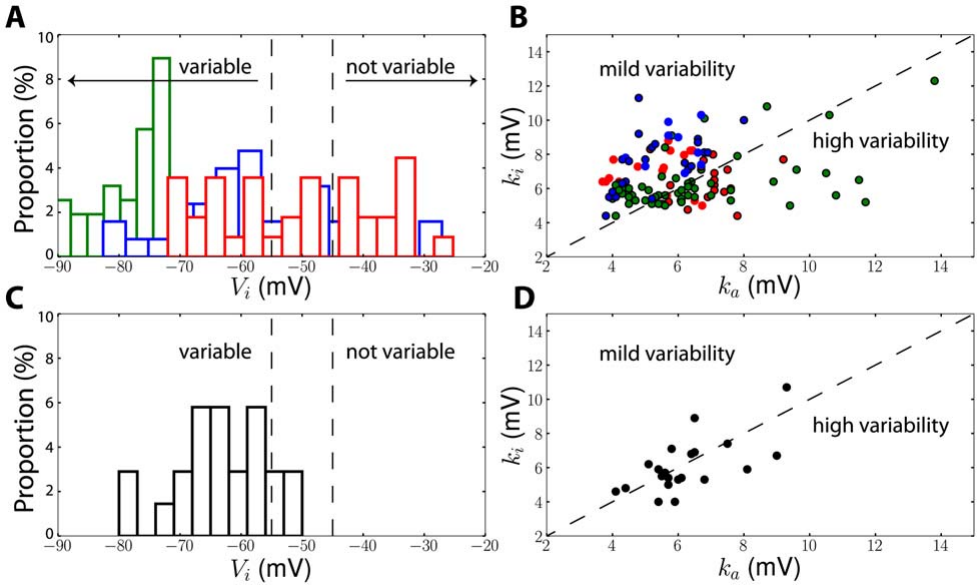
Measured properties of Na channels and threshold variability. A, Distribution of half-inactivation voltage (V_i_) of Na channels expressed in exogenous systems (from a database of 40 Na channels reported in Angelino and Brenner, 2007 [25]), including central neuron channel types (red), sensory neuron channel types (blue) and muscular channel types (green). Assuming a minimum spike threshold between −55 mV and −45 mV (dashed lines), channels on the left have variable threshold while channels of the right have a constant threshold. B, Inactivation (k_i_) vs. activation slope (k_a_) for the same dataset. Channels with V_i_<−50 mV (variable threshold) are indicated by a black contour. These channels have high threshold variability when k_a_>k_i_ (right of the dashed line). C, Distribution of V_i_ for Na channels expressed in central neurons *in situ* (see Table S1). The threshold should be variable in most cases. D, Inactivation (k_i_) vs. activation slope (k_a_) for the same dataset. High threshold variability is predicted in about half cases.

Figure 2C-E illustrates case 2 in a single-compartment model with fluctuating inputs (note that the membrane potential can exceed the threshold without triggering a spike because spike initiation is not sharp, unlike in real cortical neurons and in multicompartmental models; see the discussion in [23]). We started by examining these conditions in the dataset collected in the literature by Angelino and Brenner [25] about the properties of the 9 Nav1 channel types. These properties were obtained from voltage clamp measurements of Na channels expressed in exogenous systems. Figure 3A shows the distribution of V_i_ in this dataset, which is rather wide (−90 mV to −25 mV). Central neuron channel types, i.e., Nav1. [1], [2], [3], [6] [27], are shown in red. Since the minimum threshold V_T_ depends on the maximal Na conductance, it cannot be deduced from channel properties alone. Considering that V_T_ should lie between −55 and −45 mV [28], a substantial part of the channels fall into the first case, i.e., constant threshold, while the rest can fall into the second (moderate threshold variability) or third case (unbounded variability), depending on whether k_a_>k_i_. Figure 3B shows that, while this latter condition is never met for channel types expressed in sensory neurons (blue dots), about half of those expressed in central neurons (red) and muscles (green) satisfy k_a_>k_i_. Thus, it seems that all three cases occur in similar proportions for channel types expressed in central neurons.

However, not all Na channels are involved in spike initiation. In particular, in central neurons, spike initiation is mediated by Nav1.6 channels while Nav1.2 channels are involved in axonal backpropagation [8]. This first dataset contained only 4 Nav1.6 channels, for which V_i_<−50 mV in all cases (−61±8.4 mV), suggesting significant threshold variability, but this is a small sample. Besides, this first dataset was somewhat artificial, because channels, some of which had mutations, were artificially expressed in an exogenous system, which might alter their properties. Therefore we looked at a second dataset, consisting of *in situ* measurements in intact central neurons that we collected in the literature (see Table S1). These measurements may combine the properties of several channel types expressed at the same site, e.g. Nav1.1, Nav1.2, or Nav1.6. In some of these studies, the threshold was also measured and found to be variable [8], [17], [29], [30]. In this dataset, as shown in Figure 3C, the half-inactivation voltage was always lower than −50 mV, which implies that most channels induce threshold variability (cases 2 and 3). About half of them met the condition k_a_>k_i_ (Figure 3D). Thus, in this dataset, Na inactivation induces unbounded threshold variability in about half cases and moderate variability in the other half.

### Threshold dynamics

We have shown that Na channel properties, i.e., parameters 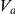, 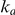, 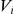,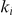, allow us to determine whether Na inactivation can make the spike threshold variable and we found that the answer is positive in central neurons. While this analysis gives an estimate of potential threshold variability, the observed variability and its properties depend on the stimulation. The instantaneous value of the spike threshold depends on the value of the inactivation variable *h* through the following formula [23]: 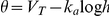. We now assume that *h* evolves according to a standard Hodgkin-Huxley equation with first order kinetics:
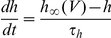
where 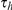 is the inactivation time constant. By differentiating the threshold equation and substituting the differential equation for h, we obtain a differential equation for 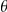 as function of the membrane potential (see Text S1 A), which can be approximated by:
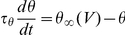
with 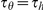. To simplify the calculations, we assume in the following that the inactivation time constant 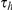 does not vary significantly with V, but we examine the effect of this voltage-dependence later. This equation describes how the threshold changes with the membrane potential, and therefore with the stimulation, and is entirely determined by Na channel properties. Since the steady-state threshold 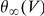 increases with V (Figure 2), it appears that the threshold adapts to the membrane potential with characteristic time 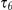. Thus, we readily see that 1) the threshold increases with the membrane potential and 2) the threshold is lower for faster depolarization, because it has less time to adapt to the membrane potential.

Before we describe threshold dynamics in more details, we need to make an important remark. As is seen in Figure 2E, which describes the dynamics of an iEIF model with fluctuating inputs, the membrane potential can exceed the threshold without triggering a spike, if the fluctuation is fast enough. This reflects the fact that spike initiation in this model, as in any biophysical single-compartment model, is not sharp: since there is no well-defined voltage threshold, what we describe as threshold variations are more accurately described as voltage shifts of the excitability curve. This makes the definition of a dynamic threshold a little ambiguous. However, spike initiation in cortical neurons is much sharper than in single-compartment models [5], because of the active backpropagation of spikes from the initiation site [6]. A direct *in vitro* measurement of the slope factor in cortical neurons (characterizing spike sharpness) gave Δ_T_≈1 mV [18] (compared to k_a_ ≈ 6 mV), meaning that spike initiation is almost as sharp as in an integrate-and-fire model. This phenomenon is well captured by multicompartmental models [8], [23] and it affects spike sharpness independently of threshold variability: in Figure 7H of ref. [23], spikes are initiated as soon as the membrane potential exceeds the dynamic threshold, which is determined according to the threshold equation. This motivates us to introduce a new model, the inactivating integrate-and-fire model (iLIF, see Methods), which is simply an integrate-and-fire model with an adaptive threshold given by the differential equation above (after a spike, the voltage is reset to the resting potential E_L_, and the threshold is increased - see Methods). This phenomenological model is not only simpler, but also seemingly more realistic than the iEIF model for the present problem, in that it reproduces both the sharpness of spike initiation and the variability of spike threshold. We use this model in the remainder of this paper.

The threshold also increases with each action potential [23] (see also Text S1 A), as was recently demonstrated *in vitro* [18]. This can be described as simple additive shift: 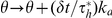, where 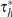 is the average value of the time constant 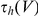 during the action potential and 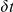 is the spike duration (typically, a few ms). If the inactivation time constant is short compared to the typical interspike interval, then this shift results in a relative refractory period, but has negligible influence on the subsequent dynamics of the model. If it is long, it results in spike-frequency adaptation and explains *in vivo* observations where the threshold was found to be inversely correlated with the previous interspike interval [13]. This phenonemon can be seen in the noise-driven iLIF model when Na inactivation is slow (not shown). In the following, we focus on the impact of fast Na inactivation.

**Figure 4:**
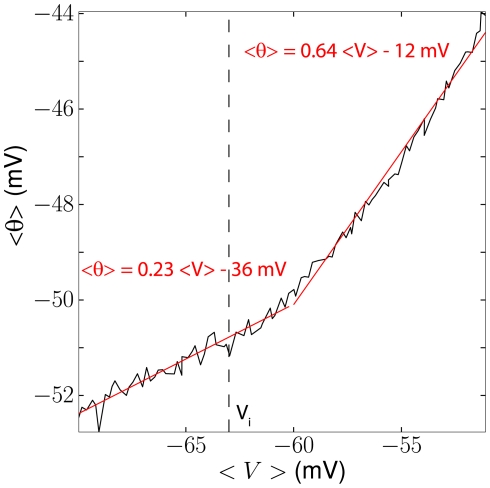
Predicted relationship between mean membrane potential and mean threshold. We simulated the iLIF model (see Methods) with a fluctuating input current. The standard deviation was fixed while the mean current was varied between trials. The mean spike threshold (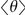) is plotted as a function of the mean membrane potential (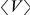). The slope of the curve is larger above half-inactivation voltage V_i_ (0.64 from linear regression, red line) than below (0.23).

Quantitatively, the relationship between average membrane potential and threshold depends on the steady-state threshold function 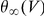. Figure 4 shows this relationship in a neuron model with adaptive threshold (defined by the dynamical equation above) and fluctuating inputs of varying mean. As expected, the average threshold increases with the average membrane potential, and the slope is steeper above half-inactivation voltage V_i_. In these simulations, the slope of the steady-state threshold curve was k_a_/k_i_ = 1, close to experimental values, but we note that the average threshold only increases as about 2/3 the average membrane potential in the depolarized region. This is because the membrane potential is very variable (about 6 mV in this figure) and therefore the threshold is not constantly in the sensitive region (V>V_i_). This is consistent with previous measurements in the visual cortex *in vivo*, where Azouz and Gray (2003) found a linear correlation with a slope of 0.5.

**Figure 5 pcbi-1001129-g005:**
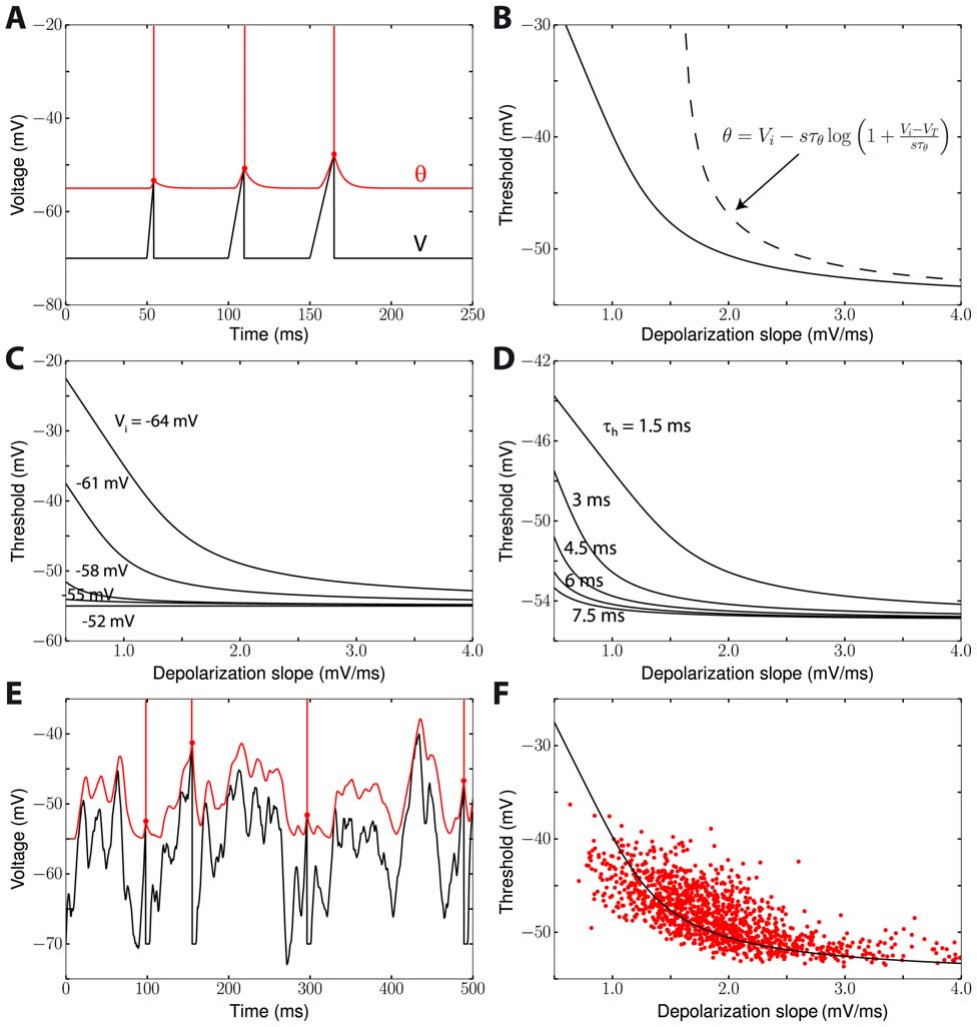
Slope-threshold relationship in the adaptive threshold model. A, The neuron is linearly depolarized with a given slope s (V(t) = E_L_+st) until the membrane potential (black) reaches threshold (red) and the neuron spikes. The intersection of the black and red traces (red dots) can be calculated (see Results). B, Threshold vs. depolarization slope (solid line) and analytical formula when k_a_ = k_i_ (dashed line). C, Slope-threshold relationship for different values of the half-inactivation voltage V_i_ (V_i_ = −63 mV in panels A,B). D, Slope-threshold relationship for different values of the inactivation time constant (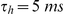 in panels A,B). E, The iLIF model is driven by a fluctuating current and we measure the slope of depolarization before each spike over a duration 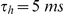 by linear regression. F, Slope-threshold relationship measured with linear regression in the noise-driven iLIF model (red dots), superimposed on the calculated relationship from panel B.

To calculate the relationship between the slope of depolarization and the threshold, we consider a linear depolarization with slope s (i.e., V(t) = V_0_+st) and calculate the intersection with the threshold 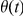 (Figure 5A). By linearizing the steady-state threshold 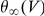 as previously described, we find that the slope s and the threshold 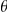 are related by the following equation (see Methods):
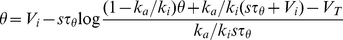



Unfortunately, this implicit equation does not give a closed formula for 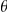 as a function of s, except when 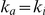:
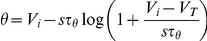



In this particular case, the threshold diverges to infinity at 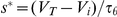, i.e., no spike is produced if the depolarization is slower than s* (Figure 5B, dashed line). This phenomenon can occur more generally when 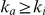 (unbounded variability, case 3) and has been observed in neurons of the cochlear nucleus [16] (where it is described as a "rate threshold"). In all cases, for large *s* (fast depolarization), the threshold 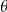 tends to V_T_, i.e., to the lowest possible threshold, and it increases for smaller *s*, i.e., slow depolarization (Figure 5B, solid line). The equations show that the slope-threshold relationship depends on the half-inactivation voltage V_i_ and on the threshold time constant 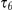 ( = 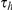). The relationship is more pronounced when V_i_ is low compared to the minimum threshold V_T_ (Figure 5C; V_T_ was −55 mV). The role of the threshold time constant can be seen as a scaling factor for slopes, i.e., the threshold depends on the product 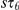 of the slope and threshold time constant. The slope-threshold relationship is more pronounced when the threshold time constant is short (Figure 5D). In experiments *in vivo*, the slope-threshold relationship was measured using linear regression on the membrane potential preceding each spike [2], [4]. We simulated the adaptive threshold model with a fluctuating input (Figure 5E) and performed a similar analysis, by calculating the depolarization slopes over a duration equal to the threshold time constant. The resulting slope-threshold relationship matches our previous calculation (which only uses Na channel properties), but with more variability (Figure 5F), as is also observed in experiments. Finally, we measured the slope-relationship in the multicompartmental model of Hu et al. [8] with fluctuating inputs, for which we previously showed that the threshold equation accurately predicted the measured threshold [23]. The slope-threshold relationship also matched our prediction (Figure S1).

### Threshold variability with fluctuating inputs

**Figure 6 pcbi-1001129-g006:**
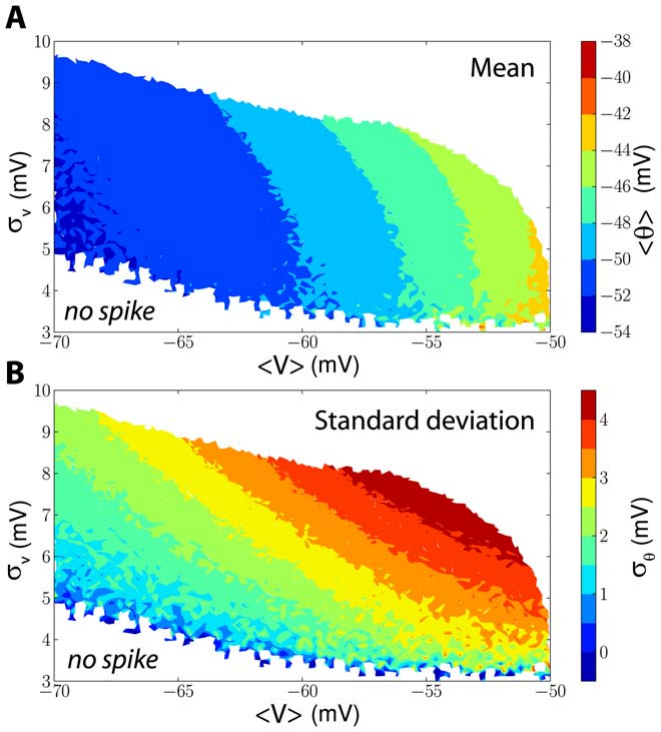
Threshold distribution as a function of membrane potential statistics. An iLIF model was stimulated by fluctuating inputs with different means and standard deviations and the threshold distribution was measured. A, Average threshold (color-coded) as a function of the mean (<V>) and standard deviation (σ_V_) of the membrane potential. The average threshold depends primarily on the average membrane potential. White areas correspond to parameter values that were not tested (top) or that elicited no spike (bottom). B, Standard deviation of the threshold as a function of membrane potential statistics. Threshold variability depends on both the average and the standard deviation of the membrane potential.

These dynamical properties of the threshold imply that the threshold should be variable for fluctuating inputs (typical of *in vivo* regimes) but not for constant DC inputs (typical of *in vitro* stimulations). More generally, it implies that the threshold distribution depends on the membrane potential distribution, as shown in Figure 6 with a neuron model with adaptive threshold driven by fluctuating inputs with different statistics. The average threshold depends mainly on the average membrane potential (Figure 6A), but the standard deviation is correlated with both the average and the standard deviation of the membrane potential (Figure 6B). This could underlie the observed difference in threshold variability between spontaneous activity (<σ> = 1.4 mV) and visual responses (<σ> = 2.3 mV) [1], because in visual responses the membrane potential is presumably both more depolarized and more variable. Interestingly, fast spiking cells showed lower threshold variability together with a lower mean threshold, which is also consistent with our results.

### Implications for synaptic integration

These results have two main implications for synaptic integration: 1) threshold adaptation reduces the impact of the input mean, relative to its variance, and 2) the negative correlation between threshold and depolarization rate shortens the timescale of synaptic integration.

#### Sensitivity to the mean and variance of inputs

**Figure 7 pcbi-1001129-g007:**
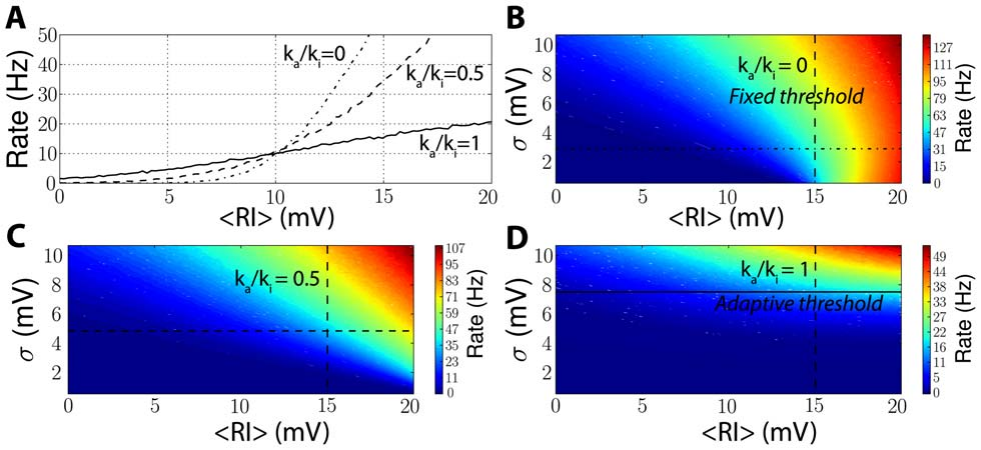
Firing rate as a function of input statistics. An iLIF model was simulated in the same way as in Figure 6, but with different values for the parameter k_a_/k_i_, which controls threshold adaptation. A, Output firing rate vs. mean input with threshold adaptation (solid line, k_a_/k_i_ = 1), with mild threshold adaptation (dashed line, k_a_/k_i_ = 0.5) and without threshold adaptation (mixed line, k_a_/k_i_ = 0). The horizontal axis is the input resistance R times the mean input <I>, i.e., the mean depolarization in the absence of spikes. The input standard deviation was chosen so that the neuron fires at 10 Hz when the mean depolarization is 10 mV. B, Firing rate (color-coded) vs. mean and standard deviation of the input, without adaptation (k_a_/k_i_ = 0). The standard deviation is shown in voltage units to represent the standard deviation of the membrane potential in the absence of spikes, i.e., 
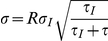
, where σ_I_ is the input standard deviation (in current units) and 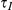 is the input time constant. The horizontal mixed line corresponds to the mixed line shown in panel A, and the vertical dashed line corresponds to the threshold for constant currents. C, Same as B, but with mild threshold adaptation (k_a_/k_i_ = 0.5). D, Same as B, but with normal threshold adaptation (k_a_/k_i_ = 1).

When V>V_i_, the steady-state threshold increases with the voltage (Figure 2A), with a slope close to 1. As a result, when the neuron is driven by a fluctuating input (such as a sum of random synaptic currents), the average threshold increases with the average membrane potential, as shown in Figure 4. Because the slope of this relationship is close to 1 (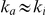), the average difference between the instantaneous value of the threshold and the membrane potential should be nearly constant above V_i_: 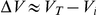. Thus, we expect that the mean of the input should have little impact on postsynaptic firing, while it should be more sensitive to its variance. Figure 7 shows the results of simulations where fluctuating currents with varying mean and variance were injected into a neuron model with adaptive threshold. When the threshold does not adapt, the output firing rate is sensitive both to the mean and the variance of the input (Figure 7A, mixed line, and Figure 7B). When the mean is above threshold (−55 mV in Figure 7), the firing rate is mostly determined by the mean. However, as threshold adaptation is increased (Figure 7A, dashed and solid lines, and Figure 7C,D), the firing rate becomes less and less sensitive to the input mean and relatively more sensitive to the variance. When threshold adaptation parameters correspond to experimentally measured properties of Na channels (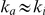), the firing rate is mostly sensitive to the input variance, although the mean input still plays a role. Thus, by maintaining a constant difference between average potential and threshold, Na channel inactivation acts as a homeostatic mechanism.

#### Timescale of synaptic integration

**Figure 8 pcbi-1001129-g008:**
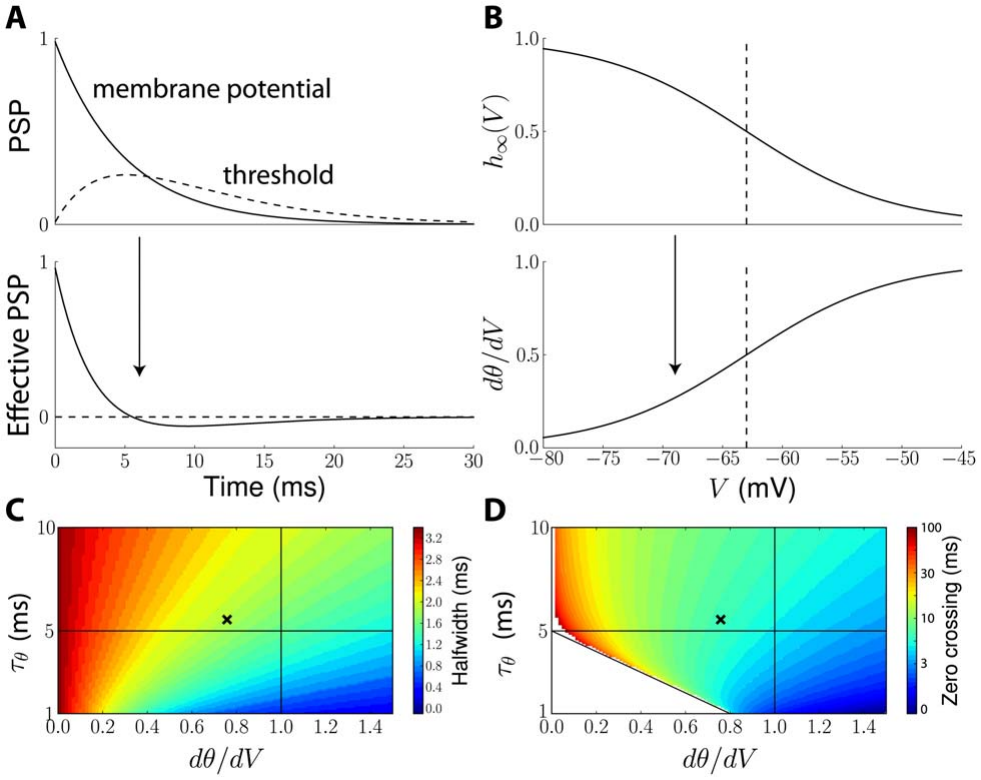
The effective postsynaptic potential. A, Top: Normalized postsynaptic potential (PSP, solid line) and threshold PSP, i.e., effect of the PSP on the threshold (dashed line). Bottom: The effective PSP is the difference between the PSP and the threshold PSP. It is briefer and can change sign. B, The effect of the PSP on spike threshold depends on how the threshold changes with voltage (dθ/dV, bottom), which depends on the membrane potential V and is determined by the Na inactivation curve (top; dashed line: half-inactivation). At high voltage, dθ/dV =  k_a_/k_i_ ( = 1 here). C, Half-width of the effective PSP (color-coded) as a function of threshold sensitivity dθ/dV and the threshold time constant 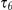. The black cross corresponds to the situation shown in panel A. The membrane time constant (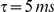) is shown by a horizontal solid line. D, Zero crossing time of the effective PSP as a function of threshold sensitivity and threshold time constant. The white triangle corresponds to parameter values where the effective PSP is always positive.

It was remarked in previous studies that the negative relationship between threshold and depolarization rate should make the neuron more sensitive to coincidences [2], [4], because depolarization is faster and thus threshold is lower for coincident inputs. We make this remark more precise by looking at *effective* PSPs, defined as the difference between the PSP and the dynamic threshold (Figure 8). Consider a neuron model in which the membrane potential is described by a sum of PSPs:
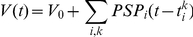
where 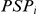 is the PSP at synapse i and 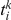 is the timing of the 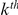 spike received at synapse i. If we approximate threshold dynamics by a linear differential equation (when V>V_i_), then the threshold 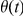 is a low-pass filtered version of 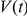:
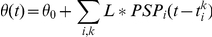
where L is a first-order low-pass filter with time constant 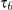 (i.e., cutoff frequency 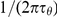), i.e.:
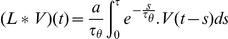
where 
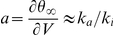
. This model with adaptive threshold is equivalent to a model with fixed threshold 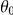, where the voltage is defined by 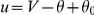, i.e., relatively to the threshold. In this equivalent model, the voltage reads:
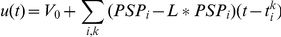



Thus, it is a linear superposition of *effective* PSPs (ePSPs), defined as the difference between the PSP and the threshold PSP (effect of PSP on threshold):
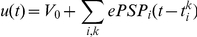
where 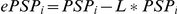 is the effective PSP at synapse *i*. This equivalent model has exactly the same form as the initial model (superposition of PSPs), the only difference being that PSPs are replaced by effective PSPs with a different shape. This is illustrated in Figure 8A.

In other words, threshold adaptation acts as a simultaneous inhibition with slower time constant (than the excitatory PSP), or as a simultaneous excitation for inhibitory PSPs. As a result, the temporal width of effective PSPs is smaller than that of PSPs, so that the timescale of synaptic integration is shorter (Figure 8A,C; see also Text S1 B for analytical calculations). Far from V_i_, i.e., when the threshold varies linearly with the membrane potential, the threshold PSP is proportional to k_a_/k_i_, which is close to 1 in experimental measurements. Closer to V_i_, the threshold PSP is proportional to dθ/dV, which lies between 0 and k_a_/k_i_ (Figure 8B). This means that threshold adaptation increases when the neuron is more depolarized, so that effective PSPs become sharper. This property is shown in Figure 8C, where the half-width of effective PSPs is seen to depend on the threshold time constant (sharper effective PSPs for shorter time constants) and on threshold sensitivity dθ/dV, i.e., indirectly on depolarization. In all cases, effective PSPs are always sharper than PSPs. For example, when the threshold time constant equals the PSP time constant and the neuron is depolarized well above V_i_ (with k_a_ = k_i_), threshold adaptation reduces the half-width of the PSP by a factor greater than 2 (intersection of the two lines in Figure 8C). In some cases, the effective PSP may change sign, as shown in Figure 8A (bottom). This occurs when the threshold time constant or the threshold sensitivity is large (Figure 8D). In the case of exponentially decaying PSPs, this condition can be analytically calculated (see Text S1 B): 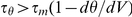. This property implies that inhibitory PSPs may trigger delayed spikes because of threshold adaptation, which we discuss below.

**Figure 9 pcbi-1001129-g009:**
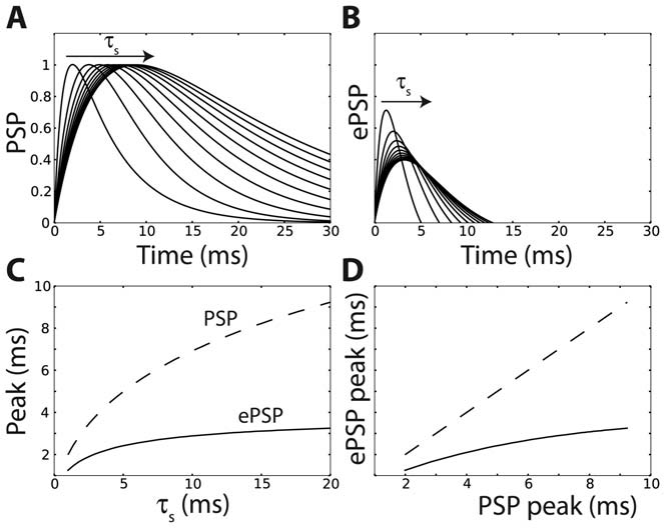
Effective postsynaptic potential with synaptic filtering. A, Normalized biexponential PSPs obtained with non-instantaneous synaptic currents (i.e., postsynaptic currents are exponentially decaying with time constant τ_s_ between 1 ms and 20 ms). B, As for exponential PSPs (Figure 8), effective PSPs (ePSPs) are narrower and change sign (only the positive part is shown). The time to peak is also shorter. Threshold adaptation parameters were 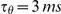 and dθ/dV = 1. C, The peak time increases with the synaptic filtering time constant τ_s_, but less rapidly for ePSPs than for PSPs. D, ePSP peak time vs. PSP peak time. Threshold adaptation makes peak times shorter and compressed.

Similar properties are seen when synaptic filtering is taken into account, that is, when the synaptic current is an exponentially decaying function rather than an instantaneous pulse (Dirac), giving biexponential PSPs (Figure 9A). As previously, effective PSPs are briefer and can change sign (Figure 9B). A new property can be observed: the peak time is shorter for ePSPs than for PSPs. This could not be seen with exponential PSPs since in that case both the PSP and the ePSP peak at 0 ms. With synaptic filtering, ePSPs peak earlier and at a smaller value. The peak time of the PSP increases with the time constant of synaptic filtering, but threshold adaptation makes ePSPs not only briefer but also less sensitive to the filtering time constant (Figure 9C,D). This phenomenon was recently demonstrated in neurons of the medial superior olive (MSO), a structure involved in the computation of interaural time differences, a cue to the azimuth of a sound source [31]. These neurons detect coincidences between inputs from the contralateral side and from the ipsilateral side. It was found that PSPs from the contralateral side peak about 500 µs later than those from the ipsilateral side, and are also shallower, which makes coincidence detection problematic (the required precision is about a few tens of microseconds). But threshold adaptation reduces the peak time of the shallower contralateral PSP, so that PSPs from both sides have similar latency. Another interesting consequence of the compression of peak times by threshold adaptation is that it also minimizes the impact of dendritic propagation on the effective latency of PSPs.

**Figure 10:**
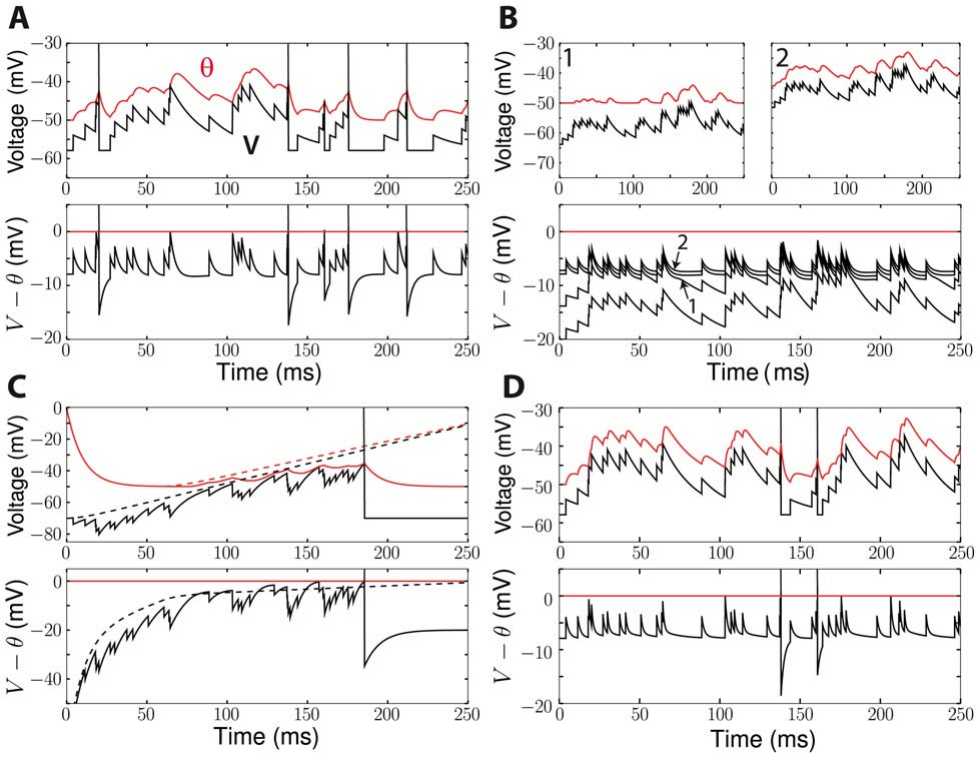
Synaptic integration with adaptive threshold. A, The iLIF model was simulated with random inputs (exponentially decaying PSPs), temporally distributed according to a Poisson process. Top: Spikes are produced when the membrane potential V (black) exceeds the threshold θ (red). Bottom: This is equivalent to a model with fixed zero threshold (red) and potential V-θ (black), which is the sum of effective PSPs. Effective PSPs are sharper than PSPs. B, Top: The threshold is more adaptive when the neuron is depolarized (right) than near resting potential (left). Bottom: When the mean input is increased (4 different levels shown), effective PSPs become sharper and their negative part cancels the input mean (see Figure 8). C, Random inhibitory PSPs are added to a depolarizing current ramp. Without inhibitory inputs (dashed), the threshold adapts and the neuron does not spike. With inhibitory inputs (solid), the sign change in effective PSPs (see Figure 8) acts as a rebound and triggers spikes. This phenomenon is often called postinhibitory facilitation [36]. D, When the voltage dependence of the Na inactivation time constant is taken into account (see Methods), effective PSPs become sharper as the neuron is more depolarized, which implies an adaptive coincidence detection property [2].

As is illustrated in Figure 10A, the reduction of PSP width makes the neuron more sensitive to coincidences at the timescale of threshold dynamics, i.e., of Na inactivation. This property only arises when the neuron is sufficiently depolarized, i.e., when V>V_i_ (Figure 10B). In high-conductance states that are typical of *in vivo* activity [32], [33], the mean membrane potential is depolarized, typically around −60 mV, which is slightly higher than the average V_i_ in the dataset of Na channels in central neurons *in situ* (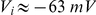; Figure 3C). Thus, neurons *in vivo* should be more sensitive to coincidences at the timescale of Na inactivation. This comes in addition to the fact that the membrane time constant is about 5 times shorter *in vivo* than *in vitro* because of increased total conductance [34], [35]. More precisely, the shape of effective PSPs depends on depolarization: as the neuron is more depolarized, the fast component of the effective PSP (which decays with time constant 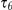) becomes more dominant, so that the neuron becomes more sensitive to fine correlations (Figure 8C).

For inhibitory PSPs (IPSPs), threshold adaptation is equivalent to simultaneous excitation with a slower time constant. Thus, in some cases, the later part of the effective PSP can be positive (Figure 8D), and therefore an IPSP can trigger a spike (Figure 10C). This phenomenon is generally called postinhibitory facilitation. It has been previously observed in different systems, and can be mediated by other mechanisms than Na inactivation [36], [37]. Figure 10C shows an example of postinhibitory facilitation due to Na inactivation, where a slow depolarization fails to trigger a postsynaptic spike but additional IPSPs do.

Finally, while we have previously ignored the voltage dependence of the time constant of Na inactivation, we show in Figure 10D how it affects synaptic integration. The time constant decreases when the neuron is depolarized above V_i_ (see Methods), which reduces the half-width of effective PSPs (Figure 8C,D). This property was termed *adaptive coincidence detection* in previous experimental studies [2].

## Discussion

Based on voltage clamp measurements of Na channel properties, we have found that Na inactivation can produce by itself large threshold variability, as observed in experiments *in vivo* [1]−[4]. Our analysis led us to a simple theoretical criterion on Na channel properties (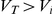 for moderate variability and 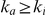 for unbounded variability). Threshold dynamics are then inherited from the dynamics of Na inactivation, which implies that the threshold adapts to the membrane potential. As a consequence, the threshold is correlated with the preceding membrane potential and inversely correlated with the depolarization rate. Both properties were observed in experiments and the quantitative relationships are close to what we predict from the properties of Na inactivation. Our analysis also provides a simple adaptive equation which describes threshold dynamics.

The criterion for large threshold variability (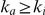) depends on the precise values of the half-activation (k_a_) and half-inactivation voltages (k_i_), obtained from Boltzmann fits. However, the relevant voltage range for these fits is the spike initiation range, and reported experimental values generally correspond to fits over the entire voltage range. This could contribute a significant measurement error in these values, as we previously showed [23]. Another potential source of error is the overlap between activation and inactivation. If the inactivation time constant is very short (comparable to the activation time constant), then voltage-clamp measurements tend to overestimate k_a_ [23]. Thus, there is some uncertainty about the precise value of k_a_/k_i_ in Na channels.

One consequence of threshold adaptation is to reduce the sensitivity of neurons to their mean input, and to make them more sensitive to fluctuations. *In vitro*, Arsiero et al. [38] indeed observed that pyramidal cells of the prefrontal cortex were very sensitive to the variance of their inputs, even when the mean was high. *In vivo*, Ringach and Malone [39] described the responses of neurons of the primary visual cortex as linear filtering of the visual input followed by (stochastic) spiking when a threshold was exceeded. They found that the threshold (defined on an abstract variable) adapted to the input statistics, so that neurons responded only to positive fluctuations above the mean.

Threshold adaptation implies that a presynaptic spike has an effect on both the membrane potential (the classical PSP) and the spike threshold. We defined an *effective PSP* by subtracting the threshold effect from the PSP. Thus, a neuron model with adaptive threshold where the membrane potential is a sum of PSPs is equivalent to a model with fixed threshold where the potential is a sum of effective PSPs. We found that effective PSPs were briefer than PSPs, which makes neurons more sensitive to input correlations at the timescale of Na inactivation. The effect of threshold adaptation can be understood as simultaneous inhibition for EPSPs and simultaneous excitation for IPSPs. These effective PSPs become briefer as the neuron is more depolarized, which can be seen as a form of adaptive coincidence detection: as the neuron is more depolarized, it requires more precisely coincident inputs to fire. This suggests that the effective integration time constant of neurons might be even shorter *in vivo* than expected from conductance measurements [34] because neurons are significantly depolarized in high conductance states [33]. A similar sharpening effect was recently found with Kv1 channels in neurons of the medial superior olive (MSO) [40]; a linear treatment of temporal sharpening by active conductances along dendrites was also recently done [41] (although independently of threshold properties).

Although Na channel inactivation can account for all the properties that have been experimentally observed, other mechanisms could potentially contribute to threshold variability: somatic measurement when spikes are initiated in the axon, channel noise and other ionic mechanisms. We discuss below these alternative mechanisms and evaluate whether they may account for threshold adaptation.

### Remote spike initiation

A recent debate about the validity of the Hodgkin-Huxley model for cortical neurons has highlighted the fact that, for central neurons, spikes are initiated in the axon while *in vivo* measurements of the spike threshold were done at the soma, which could be an artifactual cause of threshold variability [5]-[7]. However, it is unclear whether distal initiation could account for the inverse correlation between the threshold and the preceding slope of depolarization.

**Figure 11:**
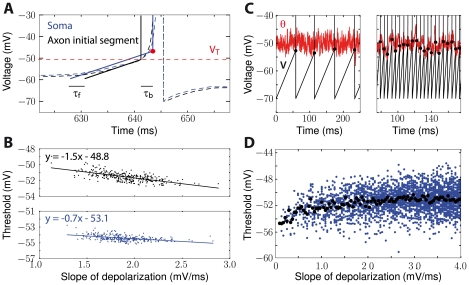
Effect of distal spike initiation and channel noise on the slope-threshold relationship. A, Illustration of the effect of depolarization slope *s* on somatic spike onset. In cortical neurons, spikes are initiated in the axon initial segment (AIS, black), then backpropagated to the soma (blue). Somatic depolarization is propagated forward to the spike initiation site in the axon with delay 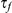. A spike is initiated in the axon when the threshold V_T_ is reached (dashed red line). The spike is backpropagated to the soma with delay 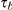. During time 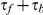, the somatic voltage has increased by 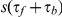 and the spike onset is seen higher (red dot). B, Slope-threshold relationship in the multicompartmental model of Yu et al. (2008) [7] with fluctuating inputs (mean 0.7 nA, standard deviation 0.2 nA, time constant 10 ms), measured at the AIS (top) and at the soma (bottom). As expected, the slope-threshold relationship is less pronounced at the soma than at the AIS. C, The effect of channel noise is modeled by a stochastic threshold (red; 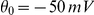, 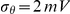, and 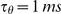) and the neuron is linearly depolarized. With slow depolarization (left), the threshold (at spike time) is lower than the average instantaneous threshold. With fast depolarization (right), the threshold distribution (at spike time) follows the distribution of θ. D, As a result, the threshold is positively correlated with the depolarization slope (blue dots: threshold vs. slope for all spikes in the simulations; black dots: average threshold for each slope).

To address this question, we consider a simplified situation where spikes are initiated in the axon hillock when the potential is above a fixed threshold V_T_ (Figure 11A). Suppose the membrane potential increases linearly in the soma (blue line) and spreads to the spike initiation site with a delay 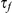 (black line). A spike is initiated when the propagated potential reaches threshold (dashed red line), and backpropagated to the soma with a delay 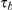. As a result, the spike “threshold” (in fact, spike onset) is higher when measured at the soma, by an amount of 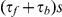, where s is the slope of depolarization. This has two consequences: 1) threshold variability is increased for fluctuating inputs, 2) the threshold is *positively* correlated with the slope of depolarization. Based on passive cable properties, the forward delay can be estimated as 
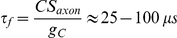
 and the backward delay as 
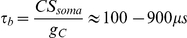
, where C is the specific membrane capacitance, 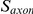 (resp. 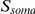) is the membrane surface of the spike initiation site (resp. soma) and 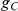 is the coupling conductance between the two sites [23]. Considering active conductances would reduce these values, but these estimations are already close to experimental measurements [42]. Thus, the total delay (forward + backward) is smaller than 1 ms.

We confirmed this reasoning by simulating the response of the multicompartmental model of Yu et al. (2008) [7] to fluctuating inputs and measuring the slope-threshold relationship both at the soma and at the axon initial segment (AIS) (Figure 11B). As we expected, we found that this relationship was more pronounced at the AIS than at the soma, meaning that the net effect of backpropagation is a positive correlation between slope and threshold. More precisely, the net effect corresponds to a total delay of 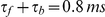 (difference between the two slopes of the linear regressions), in accordance with the estimation above. Thus, since distal spike initiation predicts the opposite relationship between depolarization rate and threshold than experimentally observed, it cannot be the dominant cause of threshold variability and cannot account for the properties of threshold dynamics.

### Channel noise

The Hodgkin-Huxley formalism describes the dynamics of the macroscopic average of many sodium channels, but individual channels have stochastic dynamics [43], [44]. It results in threshold variability which is not significantly correlated with input properties [45], [46], [43], [47], [48]. As previously, we examine whether this mechanism may account for the slope-threshold relationship in a simplified model. We consider an integrate-and-fire model with a threshold that fluctuates randomly, according to an Ornstein-Uhlenbeck process:
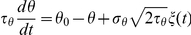
where 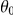 is the mean voltage threshold, 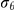 is the standard deviation of the threshold distribution, 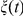 is a gaussian white noise and 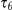 is the time constant of fluctuations (related to the time constant of Na activation).

When depolarization is very slow, spikes will be initiated lower than 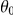 on average, because the stochastic threshold has time for many excursions below its mean, i.e., the threshold reaches the membrane potential rather than the converse (Figure 11C, left). In fact if the membrane is not depolarized (zero slope), a spike will be initiated at resting potential (although after a potentially very long time) because there is a positive probability that 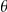 reaches that potential. On the contrary, if depolarization is very fast, spike initiation occurs at 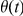, where t is near the time of depolarization, and therefore the distribution of the threshold at spike times is the same as the distribution of 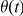 (at all times), with mean 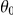 (Figure 11C, right). Therefore, the threshold is positively correlated with the slope of depolarization. We confirmed this reasoning with a numerical simulation of the model for different depolarization slopes (Figure 11D). Thus, as for distal spike initiation, channel noise produces threshold variability but induces a (weak) positive slope-threshold relationship, which is contrary to experimental findings.

### Synaptic conductances

The spike threshold increases with the total non-sodium conductance, because spike initiation requires more Na channels to be open in order to counteract a larger total conductance. Thus, fluctuating synaptic conductances could be a source of threshold variability. We previously estimated the effect of total conductance on spike threshold through the following formula [23]:
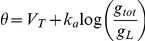
where 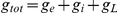 is the total conductance, including excitatory (g_e_) and inhibitory (g_i_) conductances, and we ignored the effects of Na inactivation. Threshold variability is determined by the variability of total conductance at spike time. In low-conductance states (*in vitro* or down states *in vivo*), spikes are preferentially triggered by increases in excitatory conductance g_e_ [49]. In this case, the depolarization rate is positively correlated with g_e_, and therefore with the threshold. Besides threshold variability can only be mild because the total conductance is low (relative to the leak conductance). In high-conductance states (up states *in vivo*), spikes are preferentially triggered by decreases in inhibitory conductance g_i_ [49]. In this case, the depolarization rate is negatively correlated with g_i_, and therefore with the threshold. Therefore, in high-conductance states but not in low-conductance states, the slope-threshold relationship induced by synaptic conductances is qualitatively consistent with experimental observations *in vivo*. However, with the same reasoning, the membrane potential increases when inhibition decreases and therefore, if inhibition is the main source of variability, the threshold should be negatively correlated with the preceding membrane potential, which contradicts experimental observations *in vivo*. Therefore, synaptic conductances cannot simultaneously account for the slope-threshold relationship and for the dependence on membrane potential observed *in vivo*.

### Sodium channel activation

In our analysis, we assumed that Na activation is instantaneous. Voltage clamp measurements indeed show that its time constant is only a fraction of millisecond [50], [29], [51], [52]. However, with this approximation, we might have neglected a source of threshold variability. As previously, let us examine the potential contribution of this cause of threshold variability to the slope-threshold relationship. If depolarization is slow (compared to the activation time constant), then the proportion of open channels is given by the steady-state activation curve and our analysis applies. If depolarization is very fast, fewer channels are opened than at steady state and therefore the threshold is higher. Thus, non-instantaneous activation of Na channels contributes a positive correlation between depolarization rate and threshold, contrary to experimental findings.

### Other voltage-gated channels

In the same way as synaptic conductances, voltage-gated channels may also modulate the spike threshold [23]. In particular, the delayed-rectifier potassium channel (e.g. Kv1) has been previously proposed by several authors as the source of threshold variability [2], [10], [11], [14]–[16], [21]. Indeed, a similar model to our iLIF model was previously introduced in the context of threshold accommodation by potassium channels [36]. To account for the positive correlation between membrane potential and threshold, the conductance must increase with depolarization, i.e., the activation curve must be an increasing function of the voltage. We only consider this case in this discussion. The threshold depends on the voltage-gated conductance g_K_ through the following formula:
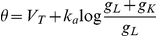
where we ignored the effect of Na inactivation. To account for significant threshold variability, two conditions must be met: 1) the maximal conductance must be large (compared to the leak) and 2) the half-activation voltage must be low enough. In this case, the spike threshold adapts to the membrane potential, which implies a positive correlation between membrane potential and threshold and a negative correlation between depolarization rate and threshold, as experimentally observed. It is also possible to differentiate the threshold equation and obtain a differential equation that describes the threshold dynamics as for Na inactivation, although it takes a different form [23]. However, there are several differences with threshold modulation induced by Na inactivation. Firstly, the threshold is always bounded by the value obtained with the maximal conductance. Secondly, the relationship between membrane potential and threshold is in general sigmoidal and can only be linear in a limited range, where the voltage is below half-activation but the conductance is still very large (the slope of this relationship is then k_a_^Na^/k_a_^K^). The impact on synaptic integration is also different, because the conductance impacts not only the threshold but also the PSPs and effective membrane time constant.

Finally, we discuss below the possible interactions of several Na channel subtypes and of slow and fast Na inactivation.

### Inactivation with several sodium channel subtypes

We assumed that a single Na channel type (e.g. Nav1.6) was present. It is possible to extend our analysis to the case of multiple subtypes. Suppose the Na current is made of two components corresponding to two channel types:
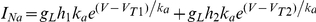



To simplify, we assumed that the two channels have the same activation Boltzmann factor k_a_, which is not unreasonable. Then the Na current can be equivalently expressed as: 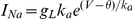
where: 
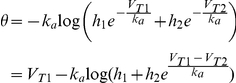



In other words, when several subtypes are present, inactivation in the threshold equation is replaced by a linear combination of inactivation variables of all subtypes. For example, Nav1.2 and Nav1.6 are both found in the axon initial segment [8], and Nav1.2 channels activate and inactivate at more depolarized potentials than Nav1.6 [53]. According to the threshold equation above, at hyperpolarized voltages, threshold modulation should be mainly determined by Nav1.6 (the inactivation variable h_2_ for Nav1.2 is less voltage-dependent and its threshold is higher); at more depolarized voltages (assuming the threshold has not been reached), Nav1.6 channels inactivate (h_1_≈0) and threshold modulation is then determined by Nav1.2 channels. Note however that with several channel subtypes, it is not possible to express threshold dynamics as a single kinetic equation for 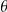 anymore (without the use of the hidden variables h_1_ and h_2_).

### Slow sodium channel inactivation

In the present study, we focused on fast Na inactivation. We have briefly mentioned that the threshold equation applies when Na inactivation is slow, and implies that the threshold increases after each spike, which induces a negative correlation between threshold and preceding inter-spike interval. This effect is expected, but it gets more interesting when the interaction between slow and fast components is considered. One way to model this interaction is to consider two Na currents, as in the previous section. But since inactivation in the same channel can show slow and fast components, it might be more relevant to include this interaction in the gating variables. The simplest way is to consider these components as independent gating processes, that is:
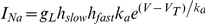
where the gating variables h_slow_ and h_fast_ have slow and fast dynamics, respectively [54], [55]. Since the interaction is multiplicative for the Na current, it is additive for the threshold:
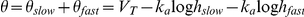



In this case, it is possible to write a kinetic equation for each component of the threshold (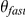 and 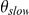), in the same way as before (note that 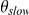 increases after each spike, whereas this effect can be neglected for 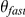 since its impact on subsequent spikes is negligible). Here, the effect of slow inactivation can be thought of as a slow change of an effective minimal threshold 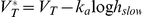 with firing activity. Interesting interactions appear because, as we have seen, threshold variability depends on the value of that minimal threshold (relative to V_i_). Suppose for example that V_T_<V_i_. At low firing rates (when interspike intervals are larger than the slow inactivation time constant), 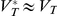 and the threshold is not variable. If the firing rate is high enough, then 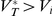 and the threshold becomes variable with fast inactivation. In the same way, the time constant of synaptic integration should be larger at low rates than at high rates. Thus, slow inactivation controls threshold modulation by fast inactivation.

In summary, many mechanisms may contribute to the variability of the spike threshold, but only two can account for its observed adaptive properties: Na inactivation and adaptive conductances (most likely K channels). Although threshold dynamics is qualitatively similar for both mechanisms, they can be distinguished by the fact that Na inactivation has no subthreshold effect on the membrane potential. Specifically, if the threshold is mainly modulated by adaptive conductances, then we can make two predictions:

1. The relationship between membrane potential and threshold should be determined by the I-V curve in the region where Na channels are closed: 
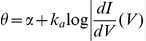
, where 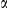 is a constant, and the I-V curve should be highly nonlinear (this derives from the threshold equation above and the fact the total conductance is dI/dV).
2. The effective membrane time constant 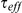 (as measured e.g. by the response to current pulses) should be inversely correlated with the threshold, through a similar formula: 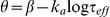, because 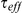 is inversely proportional to the total conductance.

In a few experimental studies, the application of α-dendrotoxin, a pharmacological blocker of low-voltage-activated potassium channels, greatly reduces threshold variability [16], which suggests a strong role for these channels in threshold adaptation. Our results suggest an alternative interpretation of these observations. The application of a blocker reduces the total conductance, which also reduces the minimum threshold V_T_ (see the threshold equation with voltage-gated channels), possibly below half-inactivation voltage V_i_, where there is no threshold adaptation due to Na inactivation. Thus, it could be that threshold adaptation was due to Na inactivation, but that suppressing K conductances shifted the minimum threshold out of the operating range of this mechanism. This hypothesis could be tested by simultaneously injecting a fixed conductance in dynamic clamp, to compensate for the reduction in total conductance of the cell.

Although we cannot draw a universal conclusion at this point, and while it is possible that either or both mechanisms are present in different cells, we observe that Na inactivation is a metabolically efficient way for neurons to shorten and regulate the time constant of synaptic integration. Indeed, Na inactivation implies no charge movement across the membrane while K+ conductances modulate the threshold by counteracting the Na current, which implies a large transfer of charges across the membrane (Na+ inward and K+ outward) in the entire region where the threshold is variable. Recently, it was found in hippocampal mossy fibers that K+ channels open only after spike initiation, in a way that minimizes charge movements [56]. Since energy consumption in the brain is a strong evolutionary pressure [57]-[59], we suggest that Na inactivation may be the main source of threshold variability when this variability has functional benefits.

## Methods

All numerical simulations were implemented with the Brian simulator [60] on a standard PC.

### Inactivating exponential model (iEIF)

Near spike initiation, the Na current can be approximated by an exponential function of the voltage [18], [24]. If the inactivation variable *h* is not discarded (see Text S1 A), we obtain the following model (membrane equation and inactivation dynamics):
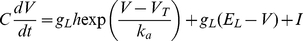
(1)

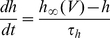
(2)where V is the membrane potential, h is the Na inactivation variable, I is the input current, C is the membrane capacitance, 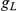 (resp. 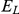) is the leak conductance (resp. the reversal potential), 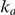 is the Na activation slope factor, V_T_ is the threshold when Na channels are not inactivated, 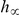 is the Na steady-state inactivation function, and 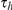 is the Na inactivation time constant, which we consider constant for simplification (except in Figure 10D). Since the model does not include K+ channels and the exponential approximation is not valid beyond spike initiation, action potentials are not realistically reproduced, but we only focus on spike initiation. We call this model iEIF (inactivating exponential integrate-and-fire model, equations (1–2)). The membrane potential is reset to 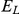 when it crosses 0 mV (h is unchanged). In Figure 2, we used 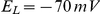, 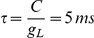 (typical membrane time constant in vivo [34]), V_T_ = −58 mV, k_a_ = 5 mV, 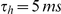, and the inactivation function was a Boltzmann function with parameters V_i_ = −63 mV and k_i_ = 6 mV.

### Adaptive threshold model and iLIF model

A very good approximation of the Na current is an exponential function of V [18], [24], [61]. The spike threshold can then be expressed with the threshold equation [23]:
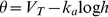
(3)where
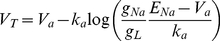
(4)is the minimum threshold, i.e., when Na channels are not inactivated (h = 1). By differentiating the threshold equation and substituting the differential equation for h, we obtain a differential equation for 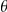 as function of the membrane potential (see Text S1), which can be approximated by:
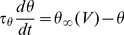
(5)with 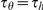, where 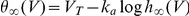 is the steady-state threshold, which can be approximated by a piecewise linear function (see Text S1):
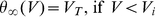
(6)

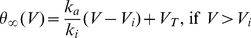
(7)


We refer to the differential equation of 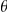 together with the expression of 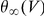 above as the *adaptive threshold model*. In simulations, we used this model with a passive membrane equation:
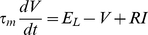
(8)where R is the membrane resistance and I is the input current, and a spike is produced when 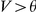. The membrane potential is then reset to E_L_. Refractoriness is implemented either by maintaining V at resting potential for 5 ms (Figure 10) or by increasing the threshold 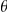 by 3.6 mV (Figures 4, 6–8), corresponding to a spike duration of 3 ms and k_a_ = 6 mV (see Text S1 A, effect of output spikes on threshold). We call this model iLIF (inactivating leaky integrate-and-fire model, equations (5–8)). In Figure 10 we used 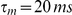 and Na parameters from a recent study of the role of Na inactivation in the temporal precision of auditory neurons [17]: 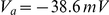; 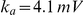; 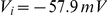; 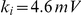. For Figures 4–8, we used V_T_ = −55 mV, V_i_ = −63 mV (average value in the *in situ* dataset), 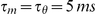. Unless otherwise specified, we chose k_a_/k_i_ = 1 (average in the dataset: 1.05).

In Figure 10D, the time constant of Na inactivation is voltage-dependent, as in [17]:
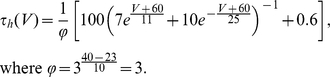



### Fluctuating inputs

Fluctuating inputs (Figures 2C–E, 6–10) were generated according to Ornstein-Uhlenbeck processes: 
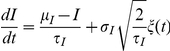
where 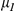 is the mean, 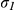 is the standard deviation, 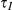 is the autocorrelation time constant, and 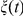 is a gaussian white noise of zero mean and unitary variance. We chose 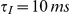 in Figure 2 and 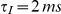 in other figures.

### Empirical threshold measurement

To measure spike onset in models with no explicit threshold (Figures 2, 10, 11), we used the first derivative method [62], which consists in measuring the membrane potential V when its derivative dV/dt crosses an empirical criterion 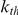. Since the input is not controlled, it measures spike onset and is an overestimate of the spike threshold. These two quantities can be related in simple models [23].

### Slope-threshold relationship

To calculate the relationship between the slope of depolarization and the threshold, we consider a linear depolarization with slope s: V(t) = st, and we calculate the intersection with the threshold 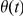 (Figure 5A), described by the adaptive threshold model. By integrating the dynamic threshold equation, we find that when 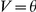 (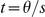), the threshold is implicitly determined by the following equation:
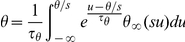



For low values of s, this equation may have no solution (i.e., the neuron does not spike). Using the piecewise linear approximation of the steady-state threshold, we obtain:
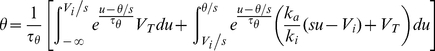
which simplifies to:
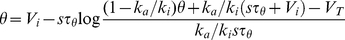



This is also an implicit equation for 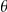, but it can be easily (numerically) calculated with a nonlinear solver. A closed formula can be obtained in the case when 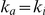:
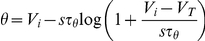



## Supporting Information

Figure S1 Slope-threshold relationship in the multicompartmental model of Hu et al. (2009), measured with linear regression over 5 ms (black dots), superimposed on the calculated relationship (red dashed line), using the Na channel properties of the model (as in Platkiewicz and Brette, 2010, Fig. 8H).
(0.17 MB PDF)

Table S1 Properties of Na channels of central neurons *in situ*.
(0.16 MB PDF)

Text S1 Impact of sodium channel inactivation on spike threshold dynamics and synaptic integration.
(0.53 MB PDF)
