# Supplementary material for: Impact of Fast Sodium Channel Inactivation on Spike Threshold Dynamics and Synaptic Integration
Source: PLoS Comput Biol. 2011 May 5;7(5):e1001129. doi: 10.1371/journal.pcbi.1001129 (PMC3088652; doi:10.1371/journal.pcbi.1001129)
Supplement: Figure S1 — Slope-threshold relationship in the multicompartmental model of Hu et al. (2009), measured with linear regression over 5 ms (black dots), superimposed on the calculated relationship (red dashed line), using the Na channel properties of the model (as in Platkiewicz and Brette, 2010, Fig. 8H). (0.17 MB PDF) [file pcbi.1001129.s001.pdf]

Supporting Information - Figure S1

# Impact of sodium channel inactivation on spike threshold dynamics and synaptic integration

Jonathan Platkiewicz[1,2] and Romain Brette[1,2] (romain.brette@ens.fr)
[1]Laboratoire Psychologie de la Perception, CNRS and Université Paris Descartes, Paris, France, and
[2]Département d'Etudes Cognitives, Ecole Normale Supérieure, Paris, France

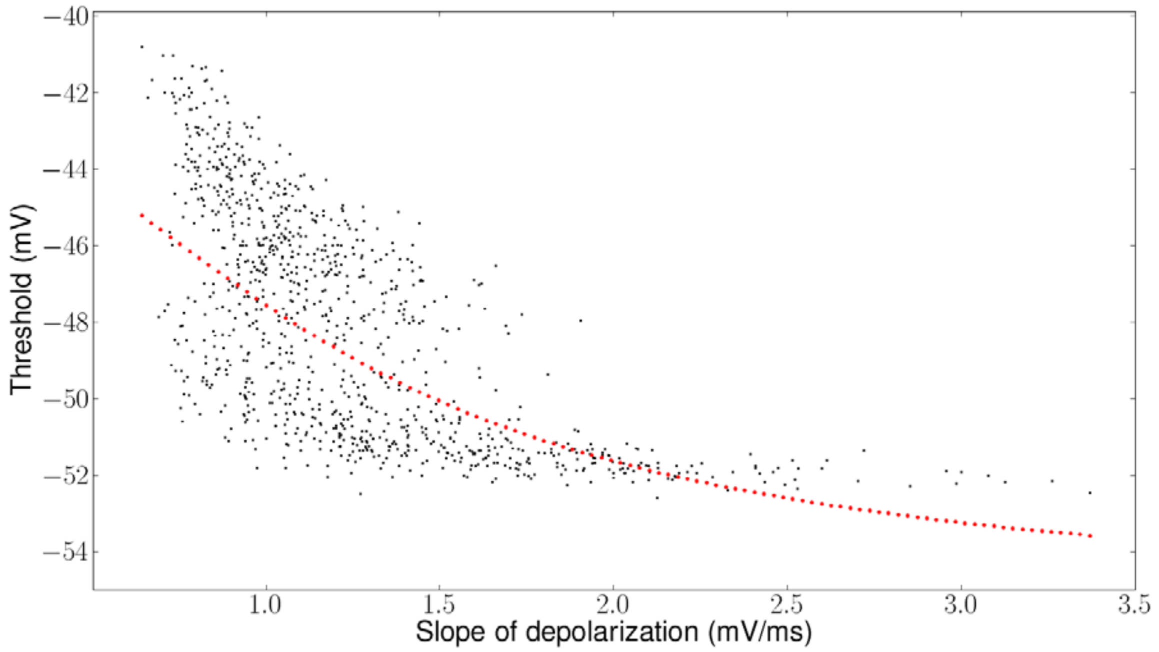


**Figure S1.** Slope-threshold relationship in the multicompartmental model of Hu et al. (2009), measured with linear regression over 5 ms (black dots), superimposed on the calculated relationship (red dashed line), using the Na channel properties of the model (as in Platkiewicz and Brette, 2010, Fig. 8H).

<u>References</u>

Platkiewicz J, Brette R (2010) A threshold equation for action potential initiation. PLoS Comput Biol 6(7): e1000850. doi:10.1371/journal.pcbi.1000850.
Hu W, Tian C, Li T, Yang M, Hou H, et al. (2009) Distinct contributions of Na(v)1.6 and Na(v)1.2 in action potential initiation and backpropagation. Nat Neurosci 12: 996–1002
