# Supplementary material for: Impact of Fast Sodium Channel Inactivation on Spike Threshold Dynamics and Synaptic Integration
Source: PLoS Comput Biol. 2011 May 5;7(5):e1001129. doi: 10.1371/journal.pcbi.1001129 (PMC3088652; doi:10.1371/journal.pcbi.1001129)
Supplement: Table S1 — Properties of Na channels of central neurons in situ. (0.16 MB PDF) [file pcbi.1001129.s002.pdf]

Impact of sodium channel inactivation on spike threshold dynamics and synaptic integration

Jonathan Platkiewicz[1,2] and Romain Brette[1,2] (romain.brette@ens.fr)
[1]Laboratoire Psychologie de la Perception, CNRS and Université Paris Descartes, Paris, France, and [2]Département d'Etudes Cognitives, Ecole Normale Supérieure, Paris, France

Table S1 - Properties of Na channels of central neurons *in situ*.

Reference	Va (mV)	ka (mV)	Vi (mV)	ki (mV)	ka/ki	Area	Cellular type	Developmental stage	Recording site	Animal
**Huguenard et al. (1988)[1]**	-30,6	5,1	-65	6,2	0,82	neocortex	pyramidal	mature	soma	rat
	-39,4	5,4	-62,6	5,9	0,92			young		
	-30,4	5,6	-68,4	5,7	0,98			immature		
	-34,1	5,4	-62	5,9	0,92		putative interneuron	mature		
	-35,2	5,6	-66,4	5,6	1,00			young		
	-26	5,5	-64,4	5,5	1,00			immature		
**Martina and Jonas (1997)[2]**	-8,82	9	-58,3	6,7	1,34	hippocampus - CA1	basket	P9 - 16	soma	rat
	-7,2	9,3	-62,9	10,7	0,87		pyramidal			
**Colbert and Pan (2002)[3]**	-31,6	6,8	-66	5,3	1,28	neocortex - layer 5	pyramidal	P14 - 24	soma	rat
	-38,4	6	-69	5,3	1,13				soma	
	-30,1	8,1	-66	5,9	1,37				axon initial segment	
**Baranauskas and Martina (2006)[4]**	-33,8	6,4	-72	6,8	0,94	prefrontal cortex	pyramidal	4 - 7 weeks old	soma	rat
**Mercer et al. (2007)[5]**	-28,5	5,7	-53,7	5	1,14	basal ganglia - globus pallidus		P16 - 22		mice
**Kole et al. (2008)[6]**	-21,7	6,5	-54,5	8,9	0,73	somatosensory cortex	pyramidal	2 - 4 weeks	soma	rat
	-31,1	6,5	-60,8	6,9	0,94				axon initial segment	
	-29,2	6,1	-58,7	5,4	1,13				axon initial segment depolymerized	
**Royeck et al. (2008)[7]**	-29,8	5,9	-50	4	1,48	hippocampus - CA1	pyramidal	P17 - 21	soma	mice
	-25	5,4	-50	4	1,35					mutant mice (deficient for Nav1.6 subunits)
**Hu et al. (2009)[8]**	-29,7	5,8	-67	7,1	0,82	prefrontal cortex	pyramidal	P16 - 20	soma	rat
	-43,9	5,7	-80	5,4	1,06				axon initial segment	
**Kuba and Ohmori (2009)[9]**	-39,5	4,4	-57,5	4,8	0,92	nucleus magnocellularis	high/middle-frequency auditory relay	P3 - 8	soma	chick
	-38,6	4,1	-57,9	4,6	0,89		low-frequency auditory relay			
**Scott et al. (2010)[10]**	-30,9	7,5	-77,4	7,4	1,01	medial superior olive	principal	P16 - 19	soma	mongolian gerbil
Mean	***-30,2***	***6,2***	***-63,1***	***6***	***1,05***					

References

1. Huguenard, J.R., Hamill, O.P. & Prince, D.A. Developmental changes in Na+ conductances in rat neocortical neurons: appearance of a slowly inactivating component. *J Neurophysiol* **59**, 778-795 (1988).
2. Martina, M. & Jonas, P. Functional differences in Na+ channel gating between fast-spiking interneurones and principal neurones of rat hippocampus. *J Physiol* **505**, 593-603 (1997).
3. Colbert, C.M. & Pan, E. Ion channel properties underlying axonal action potential initiation in pyramidal neurons. *Nat Neurosci* **5**, 533–538 (2002).
4. Baranauskas, G. & Martina, M. Sodium currents activate without a Hodgkin-and-Huxley-type delay in central mammalian neurons. *J Neurosci* **26**, 671–684 (2006).
5. Mercer, J.N., Chan, C.S., Tkatch, T., Held, J. & Surmeier, D.J. Nav1.6 sodium channels are critical to pacemaking and fast spiking in globus pallidus neurons. *J Neurosci* **27**, 13552–13566 (2007).
6. Kole, M.H.P. et al. Action potential generation requires a high sodium channel density in the axon initial segment. *Nat Neurosci* **11**, 178–186 (2008).
7. Royeck, M. et al. Role of axonal NaV1.6 sodium channels in action potential initiation of CA1 pyramidal neurons. *J Neurophysiol* **100**, 2361–2380 (2008).
8. Hu, W. et al. Distinct contributions of Na(v)1.6 and Na(v)1.2 in action potential initiation and backpropagation. *Nat Neurosci* **12**, 996–1002 (2009).
9. Kuba, H. & Ohmori, H. Roles of axonal sodium channels in precise auditory time coding at nucleus magnocellularis of the chick. *J Physiol* **587**, 87–100 (2009).
10. Scott, L.L., Mathews, P.J. & Golding, N.L. Perisomatic voltage-gated sodium channels actively maintain linear synaptic integration in principal neurons of the medial superior olive. *J. Neurosci* **30**, 2039-2050 (2010).
