# Supplementary material for: Impact of Fast Sodium Channel Inactivation on Spike Threshold Dynamics and Synaptic Integration
Source: PLoS Comput Biol. 2011 May 5;7(5):e1001129. doi: 10.1371/journal.pcbi.1001129 (PMC3088652; doi:10.1371/journal.pcbi.1001129)
Supplement: Text S1 — Impact of sodium channel inactivation on spike threshold dynamics and synaptic integration. (0.53 MB PDF) [file pcbi.1001129.s003.pdf]

Impact of sodium channel inactivation on spike threshold dynamics and synaptic integration

Jonathan Platkiewicz[1,2] and Romain Brette[1,2] (romain.brette@ens.fr)
[1]Laboratoire Psychologie de la Perception, CNRS and Université Paris Descartes, Paris, France, and
[2]Département d'Etudes Cognitives, Ecole Normale Supérieure, Paris, France

Text S1

A. Derivation of the adaptive threshold model.

We recapitulate results from our previous study[1] and complement it with a derivation of the approximation of the steady-state threshold function.

We consider a membrane with only leak and Na channels, and we assume that Na activation is instantaneous (activation time constant is typically a fraction of ms[2]). In the standard Hodgkin-Huxley formalism, the membrane equation is then:

$$C\frac{dV}{dt} = g_{Na}P_a^{\infty}(V)h(E_{Na} - V) + g_L(E_L - V) + I$$

$$\frac{dh}{dt} = \frac{h_\infty(V) - h}{\tau_h(V)}$$

where V is the membrane potential, h is the Na inactivation variable, I is the input current, C is the membrane capacitance, g_L (resp. E_L) is the leak conductance (resp. the reversal potential), g_{Na} (resp. E_{Na}) is the maximal conductance (resp. reversal potential) of sodium channels, P_a^{∞} (resp. h_∞) is the Na steady-state activation (resp. inactivation) function, and τ_h is the Na inactivation time constant.

The steady-state activation curve $P_a^{\infty}(V)$ can be empirically described as a Boltzmann function:

$$P_a^{\infty}(V) = \frac{1}{1 + \exp\left(-\frac{V - V_a}{k_a}\right)}$$

where V_a is the half-activation voltage ($P_a^{\infty}(V_a) = 1/2$) and k_a the activation slope factor ($P_a^{\infty\prime}(V_a) = 1/(4k_a)$). Action potentials are initiated well below V_a (about -30 mV[3]), so that $e^{-(V-V_a)/k_a} \gg 1$ except during the spike. Similarly, E_{Na} is very high (about 55 mV), so that $E_{Na} - V$ is not very variable below threshold. We make the approximation $E_{Na} - V \approx E_{Na} - V_a$ and we obtain the following expression for the Na current:

$$I_{Na} = g_{Na}h(E_{Na} - V_a)e^{(V-V_a)/k_a} = g_L h k_a e^{(V-V_T)/k_a}$$

where $V_T = V_a - k_a \log\frac{g_{Na}}{g_L}\frac{E_{Na}-V_a}{k_a}$. This approximation is meaningful for spike initiation but not for spike shape. With a reset (ignoring inactivation and other ionic channels), we obtain the exponential integrate-and-fire model[4], which predicts the response of cortical neurons to somatic injection with good accuracy, in terms of spike timings[5-7]. In this model, V_T is the voltage threshold for constant input currents I and k_a (originally denoted Δ_T) is the slope factor, which measures the sharpness of spikes: in the limit $k_a \rightarrow 0$ mV, the model becomes a standard

integrate-and-fire model with threshold V_T (note that in a multicompartmental model, spike sharpness is no longer related to k_a). The resulting approximated membrane equation is thus:

$$C\frac{dV}{dt} = g_L h k_a e^{(V-V_T)/k_a} + g_L(E_L - V) + I$$

Finally, the inactivation variable h can be inserted in the exponential function:

$$C\frac{dV}{dt} = g_L k_a e^{\frac{V-\theta}{k_a}} + g_L(E_L - V) + I$$

where

$$\theta = V_T - k_a \log h$$

is the spike threshold (voltage threshold if all other variables are constant, i.e., it is such that F'(θ)=0, where F is the current-voltage function). We refer to this latter formula as the *threshold equation*. The steady-state value of the threshold is thus $\theta_\infty(V) = V_T - k_a \log h_\infty(V)$.

Dynamic equation for the threshold

We differentiate the threshold equation with respect to time:

$$\frac{d\theta}{dt} = -k_a \frac{1}{h}\frac{dh}{dt} == -k_a \frac{1}{h}\frac{h_\infty(V) - h}{\tau_h(V)}$$

We now express h as a function of θ using the inverse relationships: $h = e^{(\theta-V_T)/k_a}$ and $h_\infty(V) = e^{(\theta_\infty-V_T)/k_a}$:

$$\tau_h(V)\frac{d\theta}{dt} = k_a(1 - \mathrm{e}^{\frac{\theta-\theta_\infty(V)}{k_a}})$$

This differential equation is the most accurate version of the adaptive threshold model. If the threshold remains close to its steady-state value ($|\theta - \theta_\infty(V)| \ll k_a$), we can approximate the equation by:

$$\tau_\theta(V)\frac{d\theta}{dt} = \theta_\infty(V) - \theta$$

with $\tau_\theta = \tau_h$ and $\theta_\infty(V) = V_T - k_a \log h_\infty(V)$.

Linearization of the steady-state threshold

The inactivation function is a Boltzmann function with parameters V_i (half-inactivation voltage) and k_i (inactivation slope factor). For low voltages ($V \to -\infty$), $h_\infty(V)$ tends to 1 (channels are not inactivated), therefore $\theta_\infty(V)$ tends to V_T. For high voltages, $h_\infty(V) \sim e^{-\frac{V-V_i}{k_i}}$, so that $\theta_\infty(V) \approx \frac{k_a}{k_i}(V - V_i) + V_T$. This defines two linear asymptotes of the graph of θ_∞, which intersect at (V_i, V_T). Thus, θ_∞ can be approximated by the following piecewise linear function:

$$\theta_\infty(V) = V_T, \text{ if } V < V_i$$
$$\theta_\infty\,(V) = \frac{k_a}{k_i}(V - V_i) + V_T, \text{ otherwise}$$

Thus, neglecting the voltage-dependence of the inactivation time constant, we obtain the following adaptive threshold model:

$$\tau_\theta \frac{d\theta}{dt} = \begin{cases} V_T - V & \text{if } V<V_i \\ k_a/k_i(V - V_i) + V_T - V & \text{otherwise} \end{cases}$$

Effect of output spikes on threshold

The effect of previous spikes on spike threshold, which is presumably due to slow Na inactivation[8], can be understood by looking at how an action potential acts on the inactivation variable h. Typical equilibrium curves for Na inactivation $h_\infty(V)$ are Boltzmann functions with half-activation values $V_i \approx -60$ mV and Boltzmann coefficients $k_i \approx 6$ mV[3], so that $h_\infty(V)$ is close to 0 after spike initiation. Thus during the action potential, the inactivation variable relaxes to 0 according to the following equation:

$$\tau_h(V)\frac{dh}{dt} = -h$$

If we note τ_h^* the average value of the time constant $\tau_h(V)$ during the action potential and δt the spike duration (typically, a few ms), then the effect of an action potential on h is a partial reset: $h \to he^{-\delta t/\tau_h^*}$, which translates for the threshold into a shift: $\theta \to \theta + (\delta t/\tau_h^*)k_a$. This effect was recently demonstrated *in vitro*[5] and explains *in vivo* observations where the threshold was found to be inversely correlated with the previous interspike interval[8].

B. Effective postsynaptic potentials (PSPs).

We consider an exponentially decaying PSP with time constant τ, modeling the effect of a fast excitatory synapse: $PSP(t) = e^{-t/\tau}$ (the PSP is normalized; τ corresponds to the membrane time constant). The threshold PSP is defined as the increase in threshold due to this PSP. With the adaptive threshold model and when $V>V_i$, the threshold is a low-pass filtered version of the membrane potential, i.e., $L * PSP(t) = a(e^{-t/\tau} - e^{-t/\tau_\theta})$, where

$$a = \frac{\tau}{\tau - \tau_\theta}\frac{k_a}{k_i}$$

If the steady-state threshold $\theta_\infty\,(V)$ is not approximated by a piecewise linear function, then (for small PSPs), k_a/k_i should be replaced by $d\theta_\infty/dV$ (increase rate of the threshold with depolarization).

The effective PSP is then $PSP(t) - L * PSP(t) = ae^{-t/\tau_\theta} + (1-a)e^{-t/\tau}$: it has the same maximum height as the PSP (PSP(0)=1), but first decays with the faster time constant τ_θ, then with the slower time constant τ (assuming $\tau_\theta < \tau$). In some cases the effective PSP can change sign. The zero crossing time can be calculated by solving the equation $ae^{-t/\tau_\theta} + (1-a)e^{-\frac{t}{\tau}} = 0$, which gives:

$$t^* = -\frac{\tau\tau_\theta}{\tau - \tau_\theta}\log\left(1 - \frac{(\tau - \tau_\theta)k_i}{\tau k_a}\right)$$

This is defined when the following condition is met:

$$\tau_\theta > \tau\left(1 - \left(\frac{k_i}{k_a}\right)^{-1}\right)$$

When this condition is not met, the effective PSP has constant sign (i.e. positive for an excitatory PSP, negative for an inhibitory PSP).

C. Geometrical analysis of threshold dynamics.

The excitability model is a two-dimensional dynamical system for the variables V and h. A classical approach is to look at trajectories in the (V,h) phase plane. Here we show that this approach yields qualitatively similar results as the simplified approach we have presented.

We consider the excitability model and assume that the parameter values are such that the system has three equilibria, which are geometrically represented by the intersection of the two nullclines ($\frac{dV}{dt} = 0$ and $\frac{dh}{dt} = 0$, Fig. S2A). The direction of trajectories is given by the signs of dV/dt and dh/dt. From the equations, we find: $\frac{dh}{dt} > 0$ below the h-nullcline, and <0 above; $\frac{dV}{dt} > 0$ above the V-nullcline, and <0 below. It follows that all trajectories diverge from the middle equilibrium, except two trajectories, called separatrices (Fig. S2A, green). Thus, this equilibrium is a saddle point and the separatrices are a threshold line[11]: every initial condition on its left leads to a subthreshold trajectory; every initial condition on its right leads to a suprathreshold trajectory. Because of the signs of dh/dt and dV/dt, V is a decreasing function of h along these separatrices. In other words, the voltage threshold is a decreasing function of h.

Let us consider now that the system is stimulated by a pulse input. If this pulse is short and large (ideally, a Dirac), the trajectory of the system will be almost horizontal in (V,h) (Fig. S2B, dashed blue). Otherwise, the trajectory of the system will lie below this horizontal line (Fig. S2B, solid blue). As the input gets slower, the trajectory shifts downward and, because of the inclination of the separatrices, the voltage at threshold increases. This results in a negative correlation between depolarization slope and threshold, as expected.

For fluctuating inputs, the approach based on the separatrix turns out to be quantitatively less accurate that the threshold equation, as is shown in Fig. S3.

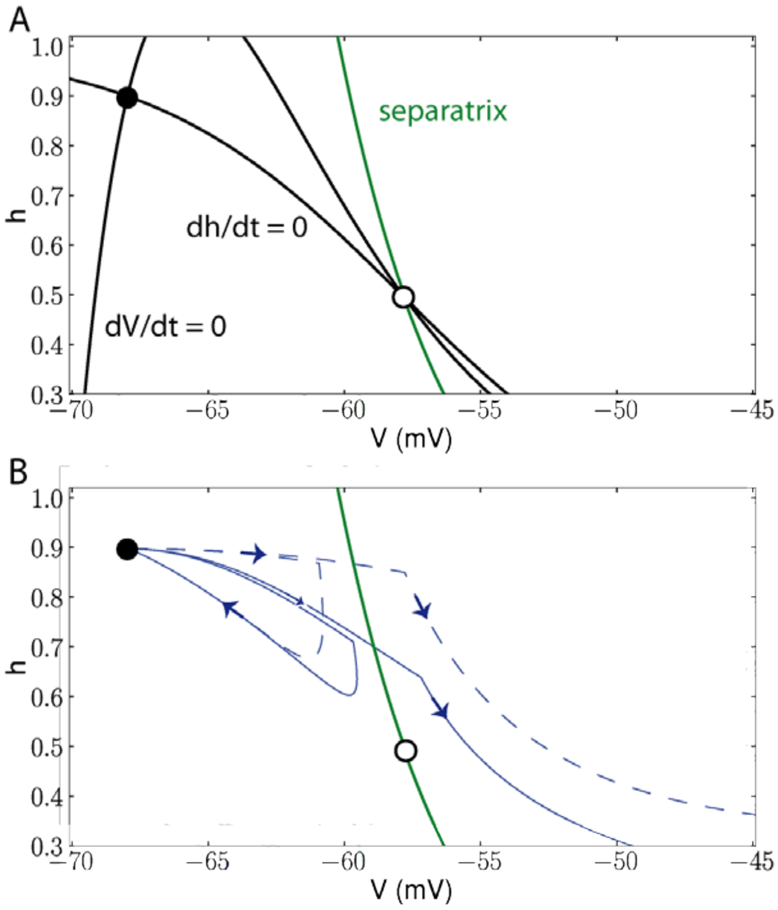

Figure S2. Threshold dynamics in the (V, h) phase plane. A, The two nullclines of the excitability model (black curves, dV/dt=0 and dh/dt=0) intersect at the resting point (black dot), which is stable, and at an unstable point (white circle). Spikes are triggered when trajectories cross the separatrix (green curve). B, Sample trajectories with pulse input currents: because of the orientation of the separatrix, spikes are triggered at lower voltages with fast depolarization (dashed) than with slow depolarization (solid).

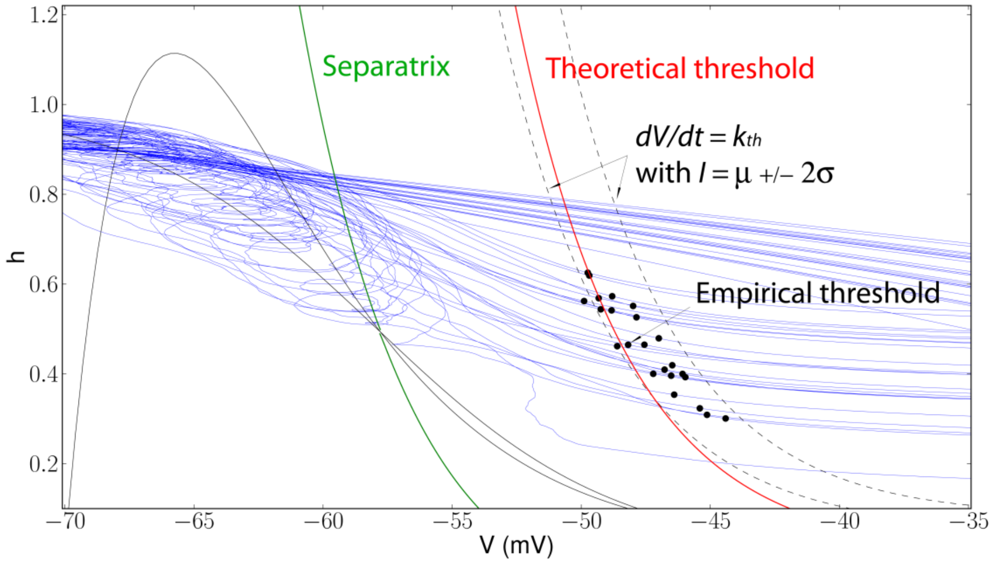

Figure S3. Trajectories in the (V,h) plane with fluctuating inputs (mean μ =0 mV, standard deviation $\sigma = 15\,mV$, autocorrelation time constant $\tau_I = 2\,ms$). In this case, our theoretical threshold prediction (red, using the threshold equation) is more accurate than the separatrix (black dots are spike onsets, measured with the empirical first derivative method, and the dashed lines represent the first derivative criterion in the model equations, with I = μ ± 2σ; the input current appears in the membrane equation and varies at spike initiation).

References

1. Platkiewicz, J. & Brette, R. A threshold equation for action potential initiation. *PLoS Comput Biol* 6(7): e1000850. doi:10.1371/journal.pcbi.1000850 (2010).
2. Baranauskas, G. & Martina, M. Sodium currents activate without a Hodgkin-and-Huxley-type delay in central mammalian neurons. *J Neurosci* **26**, 671–684 (2006).
3. Angelino, E. & Brenner, M.P. Excitability constraints on voltage-gated sodium channels. *PLoS Comput Biol* **3**, 1751–1760 (2007).
4. Fourcaud-Trocmé, N., Hansel, D., Vreeswijk, C.V. & Brunel, N. How spike generation mechanisms determine the neuronal response to fluctuating inputs. *J Neurosci* **23**, 11628–11640 (2003).
5. Badel, L. et al. Dynamic I-V curves are reliable predictors of naturalistic pyramidal-neuron voltage traces. *J Neurophysiol* **99**, 656–666 (2008).
6. Brette, R. & Gerstner, W. Adaptive exponential integrate-and-fire model as an effective description of neuronal activity. *J Neurophysiol* **94**, 3637–3642 (2005).
7. Jolivet, R. et al. The quantitative single-neuron modeling competition. *Biol Cybern* **99**, 417-426 (2008).
8. Henze, D.A. & Buzsáki, G. Action potential threshold of hippocampal pyramidal cells in vivo is increased by recent spiking activity. *Neuroscience* **105**, 121–130 (2001).
9. Rinzel & Ermentrout Analysis of neural excitability and oscillations, Methods in neuronal modeling: From synapses to networks. 135 – 169 (1989).
